# Identification of arachidonic acid metabolism-related diagnostic markers in heart failure based on bioinformatics analysis and machine learning

**DOI:** 10.3389/fcvm.2025.1625064

**Published:** 2025-12-15

**Authors:** Saiqing Chen, Chunxia Zhang, Yueting Yu

**Affiliations:** 1 Department of Emergency, Yiwu Hospital of Traditional Chinese Medicine, Yiwu, Zhejiang, China; 2 Department of Nutrition, Yiwu Hospital of Traditional Chinese Medicine, Yiwu, Zhejiang, China

**Keywords:** heart failure, arachidonic acid-related genes, biomarker, machine learning, diagnostic model

## Abstract

**Background:**

Heart failure (HF) represents the terminal phase of multiple cardiovascular conditions and is associated with significant morbidity and mortality rates. Arachidonic acid (AA), an essential fatty acid, plays a crucial role in modulating cardiovascular function under both normal and disease states. The purpose of this research was to examine how AA is related to HF, providing new perspective for individualized treatment.

**Methods:**

Transcriptomic datasets were retrieved from the Gene Expression Omnibus (GEO) database. The raw data were consolidated to identify differentially expressed genes (DEGs) and subsequently subjected to bioinformatics analysis. Gene ontology (GO) annotation and Kyoto Encyclopedia of Genes and Genomes (KEGG) enrichment analyses were performed. Signature genes were identified through Least Absolute Shrinkage and Selection Operator (LASSO) regression, Support Vector Machine-Recursive Feature Elimination (SVM-RFE), and Random Forest (RF) algorithms. Receiver Operating Characteristic (ROC) curves were generated for gene evaluation, and a nomogram was developed. An analysis of immune cell infiltration was conducted using Single Sample Gene Set Enrichment Analysis (ssGSEA), and Gene Set Enrichment Analysis (GSEA) was conducted to determine important pathways. Subsequently, we also performed drug sensitivity evaluation. Finally, the expression levels of the identified signature genes in HF samples were confirmed using qRT-PCR analysis.

**Results:**

Four characteristic genes demonstrating favorable performance in the ROC analysis. The comprehensive nomogram developed in this study exhibited enhanced clinical utility. In addition, notable variations in immune cell infiltration levels were detected, and GSEA highlighted key biological pathways.

**Conclusion:**

This investigation demonstrated a strong association between arachidonic acid-associated gene expression and heightened risk of HF, offering novel perspectives on the disease's underlying pathological processes and providing potential insights for personalized management of HF.

## Highlights

- In this study, molecular subtypes were defined based on arachidonic acid-related genes, followed by the creation of a diagnostic model.
- Four key biomarkers for heart failure with high diagnostic accuracy were recognized: GGT5, PLA2G2A, EPHX2, and CYP2J2.
- The gene-drug interaction network analysis identified 12 approved therapeutic agents associated with CYP2J2, 5 with EPHX2, and 7 with PLA2G2A.

## 1 Introduction

Heart failure (HF) represents a significant challenge in both clinical practice and public health, which is a significant cause of morbidity and mortality worldwide (1, 2). The prevalence of HF is increasing and it effects about 40 million people worldwide, which has become a key health issue (3, 4). Many cardiovascular diseases, including myocardial infarction, hypertension, valvulopathy, will lead to HF (5). Although various treatments are available, the outlook for many individuals with HF remains unfavorable (6). Within five years following a hospitalization due to HF, mortality rates vary between roughly 40% and 75% (7, 8). Additionally, China has the largest burden of HF worldwide, which has affected more than 13.7 million individuals (9, 10). Although several studies have reported that the progression of HF involves dysregulation of numerous genes and signaling pathways, the molecular mechanisms underlying HF development remain poorly characterized, thereby constraining the efficacy of current therapeutic strategies.

Polyunsaturated fatty acids (PUFAs) have traditionally been regarded as beneficial to cardiovascular health (11). Arachidonic acid (AA), a vital fatty acid and one of the most prevalent PUFAs in the human body, has recently been shown to play an important role in cardiovascular function (12–15). AA gives rise to a diverse array of metabolites, such as prostaglandins, prostacyclin, thromboxanes, hydroxyeicosatetraenoic acids, leukotrienes, lipoxins, and epoxyeicosatrienoic acids (16). The established metabolic pathways of AA include the cyclooxygenase (COX) pathway, the lipoxygenase (LOX) pathway, and the cytochrome P450 (CYP) enzyme pathway (17). These metabolites collectively constitute the “eicosanoid metabolic process” which encompasses the biosynthesis and metabolism of bioactive lipid mediators derived from AA. Eicosanoids, including prostaglandins, thromboxanes, and leukotrienes, play pivotal roles in regulating inflammation, vascular tone, platelet aggregation, and cardiac remodeling (18). Dysregulation of this process has been closely associated with the onset and progression of cardiovascular diseases, including HF. Moreover, metabolites produced by these enzymatic pathways exhibit distinct and sometimes opposing cardiovascular effects, highlighting the complex regulatory roles of eicosanoid metabolism in HF. COX-derived prostaglandins and thromboxanes regulate vascular tone, platelet aggregation, and myocardial contractility, whereas LOX-derived leukotrienes and lipoxins contribute to inflammatory and oxidative stress responses in the myocardium. Importantly, the CYP450 epoxygenase pathway converts AA into epoxyeicosatrienoic acids (EETs), which exert vasodilatory, anti-inflammatory, and cardioprotective actions (19). In addition to its vasodilatory effects, early studies demonstrate that CYP450 metabolites can also mediate negative inotropic effects on the heart. Specifically, Rastaldo R. et al. first demonstrate that CYP450 metabolites mediate bradykinin-induced negative inotropic effects, namely reducing myocardial contractility (20). Recent studies have demonstrated that EETs improve myocardial perfusion and reduce cardiac remodeling after ischemia–reperfusion injury by modulating endothelial nitric oxide synthase and calcium signaling (21, 22). Moreover, reduced EET bioavailability has been linked to endothelial dysfunction, oxidative stress, and adverse cardiac remodeling in HF (CYP2J2 Modulates Diverse Transcriptional Programs in Adult Human Cardiomyocytes). Dysregulation of these AA-derived metabolites may therefore contribute to the initiation and progression of HF. Previous evidence further supports that alterations in AA metabolism contribute to pathological processes relevant to HF, including lipid metabolic disturbances, inflammation, oxidative stress, and cardiomyocyte apoptosis (23). However, the clinical evidence supporting the prognostic value of AA metabolites in HF is still limited, warranting further investigation.

In this work, we employed bioinformatics techniques with machine learning algorithms to uncover diagnostic genes associated with HF that were significantly associated with arachidonic acid-related genes (AARGs). Additionally, we combined the risk scores of key feature genes to develop a comprehensive nomogram, enabling effective risk stratification for HF. We aimed to identify AARGs associated with HF, construct and validate a diagnostic model, and explore immune infiltration and ceRNA regulatory mechanisms.

## 2 Materials and methods

### 2.1 Data acquisition

**Figure 1:**
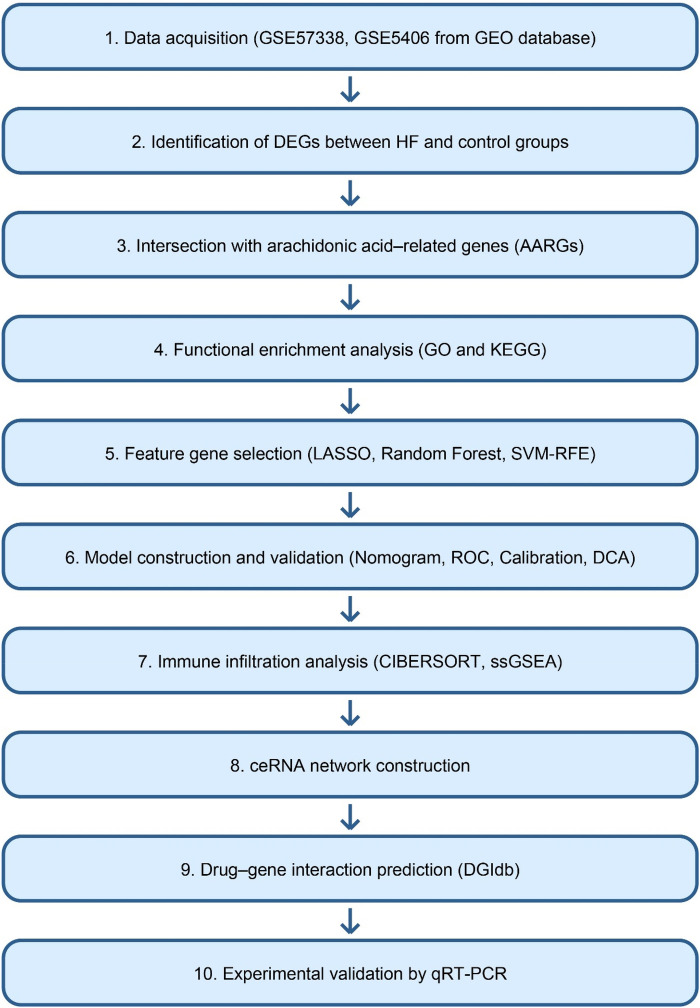
Overall workflow of the study. The schematic diagram summarizes the main analytical steps, including data acquisition, differential expression analysis, feature gene selection, model construction, immune infiltration analysis, ceRNA network construction, drug-gene interaction prediction, and experimental validation.

The expression profiling dataset GSE57338, generated by high-throughput sequencing, was obtained from the Gene Expression Omnibus (GEO) database (https://www.ncbi.nlm.nih.gov/geo/). The GSE57338 dataset comprised left ventricular myocardial tissue samples from 177 patients with end-stage HF and 136 non-failing (NF) donor hearts. According to the original publication (24), HF samples were collected from patients undergoing heart transplantation or ventricular assist device implantation due to advanced HF. NF control samples were obtained from donor hearts without any clinical or echocardiographic evidence of cardiac dysfunction. Only samples with high-quality RNA and complete clinical annotation were included, as defined by the original study. The GSE5406 dataset was downloaded as the validation set, consisting of 16 normal and 196 case samples. Both datasets were generated using Affymetrix microarray platforms (GPL570) and normalized using robust multi-array average (RMA) methods via the “affy” package (version 1.78.0) in R (version 4.2.2) before downstream analysis. To construct an HF-related gene set, HF-associated genes were retrieved from the GeneCards database (https://www.genecards.org/). These genes were intersected with the differentially expressed genes (DEGs) identified from the microarray data, yielding 365 overlapping candidates. The top 20 genes ranked by Relevance score were then selected as the final HF-related gene set for downstream analyses. A total of 58 AARGs were obtained from related literature (25). The workflow of this study was shown in Figure 1.

### 2.2 Analysis of differentially expressed genes

DEGs between the HF and normal groups in the GSE57338 dataset were identified employing the limma package (version 3.54.0). [|log_2_ fold change (FC)| > 0.5, *p* value < 0.05]. Volcano plots and heatmaps were constructed to visualize DEGs, with the heatmap displaying the top 20 DEGs based on |log_2_FC| values. The intersection of DEGs with the AARGs set was utilized to screen for differentially expressed arachidonic acid-related genes (DEAARGs). Gene Ontology (GO) analysis and Kyoto Encyclopedia of Genes and Genomes (KEGG) enrichment analyses were conducted with “clusterProfiler” (version 4.8.1) using the parameters pAdjustMethod = “BH”, qvalueCutoff = 0.05, and minGSSize = 5. The DEAARGs were integrated into a protein-protein interaction (PPI) network by utilizing STRING (version 12.0) with a confidence score cutoff greater than 0.4, and the network visualization was carried out using Cytoscape version 3.10.2.

### 2.3 Analysis of consensus cluster

Consensus clustering was performed to identify subtypes of HF based on DEAARGs using the “ConcensusClusterPlus” package (version 1.60.0) with 1,000 resamplings, a repetition fraction of 0.8, Euclidean distance, and Ward.D2 linkage. The optimal cluster number (k) was determined by evaluating the cumulative distribution function (CDF) and delta area curves.

### 2.4 Screening of diagnostic feature genes for HF based on machine learning

Least Absolute Shrinkage and Selection Operator (LASSO) regression analysis was conducted on these candidate genes utilizing the “glmnet” package (version 4.1-7) with 10-fold cross-validation to identify the optimal *λ* value. Random forest (RF) analysis was performed employing the“randomForest” package (version 4.7-1.1) with 500 trees (ntree = 500) and variable importance ranking based on MeanDecreaseGini; the top 5 genes were retained. Support Vector Machine-Recursive Feature Elimination (SVM-RFE) analysis was performed using the “e1071” and “caret” packages (version 6.0-94) with a radial-basis-function kernel and 10-fold cross-validation. Genes identified by at least two algorithms were defined as signature genes.

### 2.5 Enrichment analysis for signature genes

Gene Set Enrichment Analysis (GSEA) was performed to explore biological pathways associated with each hub gene. For each feature gene (CYP2J2, EPHX2, PLA2G2A, and GGT5), samples in the GSE57338 dataset were divided into high- and low-expression groups according to the median expression value. This grouping was exploratory in nature, designed to evaluate the relationship between the ranking performance of the whole-genome expression profile and biological pathway enrichment across different expression levels. GSEA was then conducted using the “clusterProfiler” package (version 4.8.1) based on genome-wide ranked gene expression profiles between these two groups. Specifically, first calculated statistics for each gene across two comparison groups (using the log_2_FC difference between groups as the sorting key to obtain a gene list ranked from highest to lowest log_2_FC), then performed GSEA using this sorted vector. The KEGG gene set (c2.cp.kegg.v7.5.symbols.gmt) was used as the reference database. Enrichment results with adjusted *P* < 0.05 and |Normalized Enrichment Score (NES)| > 1 were considered statistically significant.

### 2.6 Receiver operating characteristic (ROC) curve analysis

A diagnostic nomogram was developed based on a multivariable logistic regression model integrating the expression levels of four hub genes (GGT5, PLA2G2A, EPHX2, and CYP2J2) using the “rms” package (version 6.5-0) in R (version 4.2.2). The fitted model was expressed as:

$$\log\left(\frac{P}{1-P}\right)=12.5457-3.1996\times \mathrm{GGT}5-0.4444\times \mathrm{PLA}2G2A+1.4679\times \mathrm{EPHX}2+0.5504\times \mathrm{CYP}2J2$$

where P represented the predicted probability of HF, and the regression coefficients (βi) indicated the relative contribution of each gene to the model. The detailed regression coefficients and associated statistics (standard error, Wald Z, and *p*-value) were provided in Supplementary Table 1.

Model discrimination was assessed by the receiver operating characteristic (ROC) curve and area under the curve (AUC) using the “pROC” package (v1.18.4), while calibration was evaluated by the bootstrap-corrected calibration plot (1,000 resamplings) and the Hosmer–Lemeshow test. The constructed model was further validated in the external dataset (GSE5406) by calculating predicted probabilities and generating ROC curves.

### 2.7 Immune cell infiltration analysis

The immune cell abundance across normal and HF samples was performed for CIBERSORT analysis using the “IOBR” package (version 1.4.3) with the LM22 signature matrix and 1,000 permutations to calculate the relative proportions of 22 immune-related cell types. Box plots illustrating the differences in immune infiltration levels across the normal and HF samples were visualized using the CIBERSORT analysis. Samples with *P* < 0.05 were considered reliable for downstream analysis. Single-sample Gene Set Enrichment Analysis (ssGSEA) was conducted using the “GSVA” package (version 1.46.0) to evaluate immune cell infiltration profiles, with the results visualized in a heatmap and a stacked bar chart. In addition, a correlation heatmap visualizing the associations was created using the “ggcorrplot” package across the characteristic genes and the differentially infiltrated immune cells.

### 2.8 Development of competing endogenous RNA (ceRNA) network

According to the ceRNA hypothesis, long noncoding RNAs (lncRNAs), microRNAs (miRNAs), and mRNAs can interact through shared miRNA response elements, forming a post-transcriptional regulatory network. To further elucidate the molecular mechanisms underlying the disease and to identify potential therapeutic targets, we constructed an lncRNA-miRNA-mRNA ceRNA network to explore the potential regulatory mechanisms underlying the expression of the key AARGs in HF. The “multiMiR” package (version 1.20.0) was applied to identify miRNAs interacting with feature genes by screening the results from miRDB, TargetScan, and miRTarBase databases. Interaction data between lncRNAs and miRNAs were downloaded from ENCORI (https://rnasysu.com/encori/), and lncRNAs with a clipExpNum greater than 10 were selected. Visualization of the interactions was performed using the “ggsankey” package and Cytoscape version 3.10.2.

### 2.9 Drug sensitivity prediction

Drug-gene interactions were retrieved from the Drug–Gene Interaction Database (DGIdb, version 4.3.0; https://www.dgidb.org). Only approved small-molecule drugs targeting the hub genes were included. Candidate drugs were visualized using Cytoscape version 3.10.2 and further annotated for their pharmacological class and potential mechanisms.

### 2.10 Cell lines and cell culture

H9C2 cells (rat cardiomyoblasts, ATCC CRL-1446) were maintained in Dulbecco's Modified Eagle Medium (DMEM; Sigma-Aldrich) supplemented with 10% fetal calf serum and 1% streptomycin (both from Sigma-Aldrich), under standard incubation conditions of 37 °C and 5% CO_2_. HL-1 cells (mouse atrial cardiomyocytes, ATCC CRL-12197) were cultured in Claycomb medium (Sigma-Aldrich), enriched with 10% fetal bovine serum, 1% streptomycin, 1% GlutaMAX, and 0.1 mM norepinephrine (all obtained from Sigma-Aldrich), and grown at 37 °C in a humidified atmosphere containing 5% CO_2_.

### 2.11 qRT-PCR analysis

Total RNA was extracted from H9C2 cells (representing HF) and HL-1 cells (used as normal controls) using TRI Reagent (Sigma, USA). Subsequently, 2.0 μg of RNA was reverse-transcribed into cDNA with the Reverse Transcription cDNA Kit (Thermo Fisher Scientific, Waltham, MA, USA). Quantitative real-time PCR was carried out on a QuantStudio 7 Flex System (Thermo Fisher Scientific) to assess the relative mRNA expression levels. The relative expression levels in terms of fold changes of target genes were calculated by the 2^−△△CT^ method. The primers used to explore mRNA expression of hub genes were listed in Table 1.

**Table 1:** Sequences of primers for qRT-PCR.

Gene name	Primer sequences (5’-3’)
GGT5	Forward: ACCATCAACACACCCTTTGGA
	Reverse: CACTGCAGGTGAGGGGGTG
PLA2G2A	Forward: CTGTCTCCAAACAGCCTTGTG
	Reverse: CCAGGCCGTCTTGTTTGTTC
EPHX2	Forward: GAGATCACACTTTCCCAGTGGA
	Reverse: TGACTTTCTCAAAAGACGGCG
CYP2J2	Forward: TCCCATATTTCTTCACAAACAGC
	Reverse: TCCCATATTTCTTCACAAACAGC

## 3 Results

### 3.1 Analysis of differential arachidonic acid-related genes

**Figure 2:**
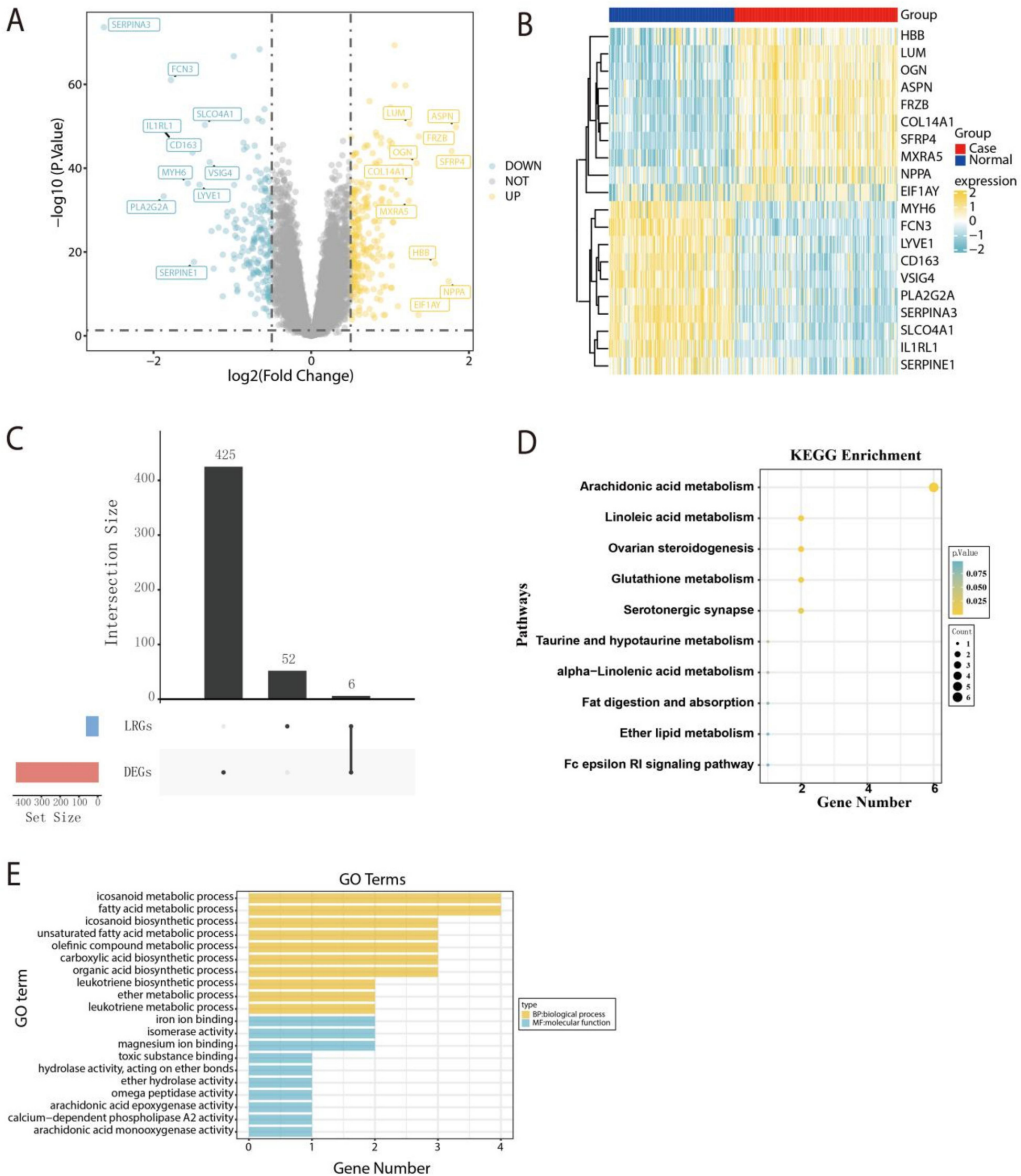
Selection and primary analysis of candidate genes associated with AA. **(A)** The volcano plot illustrating the distribution of DEGs. **(B)** The heatmap visualizing the top 20 DEGs, ranked by |log_2_FC|. **(C)** The upset plot depicts the intersection between the DEGs and AARGs gene sets. **(D)** Enrichment analysis of DEAARGs based on KEGG pathways. **(E)** Functional annotation of DEAARGs based on GO analysis.

To identify the candidate genes for HF diagnosis significantly associated with AARGs, a total of 431 DEGs were detected within the GSE57338 dataset. Among the 431 DEGs identified, 237 were up-regulated and 194 were down-regulated (Figure 2A). Figure 2B showed a heatmap displaying the top 20 DEGs ranked by |log_2_FC| values. Subsequently, we intersected the DEGs with the AARGs, resulting in 6 DEAARGs (Figure 2C). Additionally, KEGG pathway analysis indicated that DEGs were mainly involved in arachidonic acid metabolism, linoleic acid metabolism, and ovarian steroidogenesis, revealing the significantly relationship between AA and HF (Figure 2D). GO analysis revealed that DEGs were mainly associated with biological process, including participation in icosanoid metabolic process, fatty acid metabolic process, icosanoid biosynthetic process, and molecular function, including iron ion binding, isomerase activity and magnesium ion binding (Figure 2E).

### 3.2 Identification of characteristic genes for HF diagnosis

**Figure 3:**
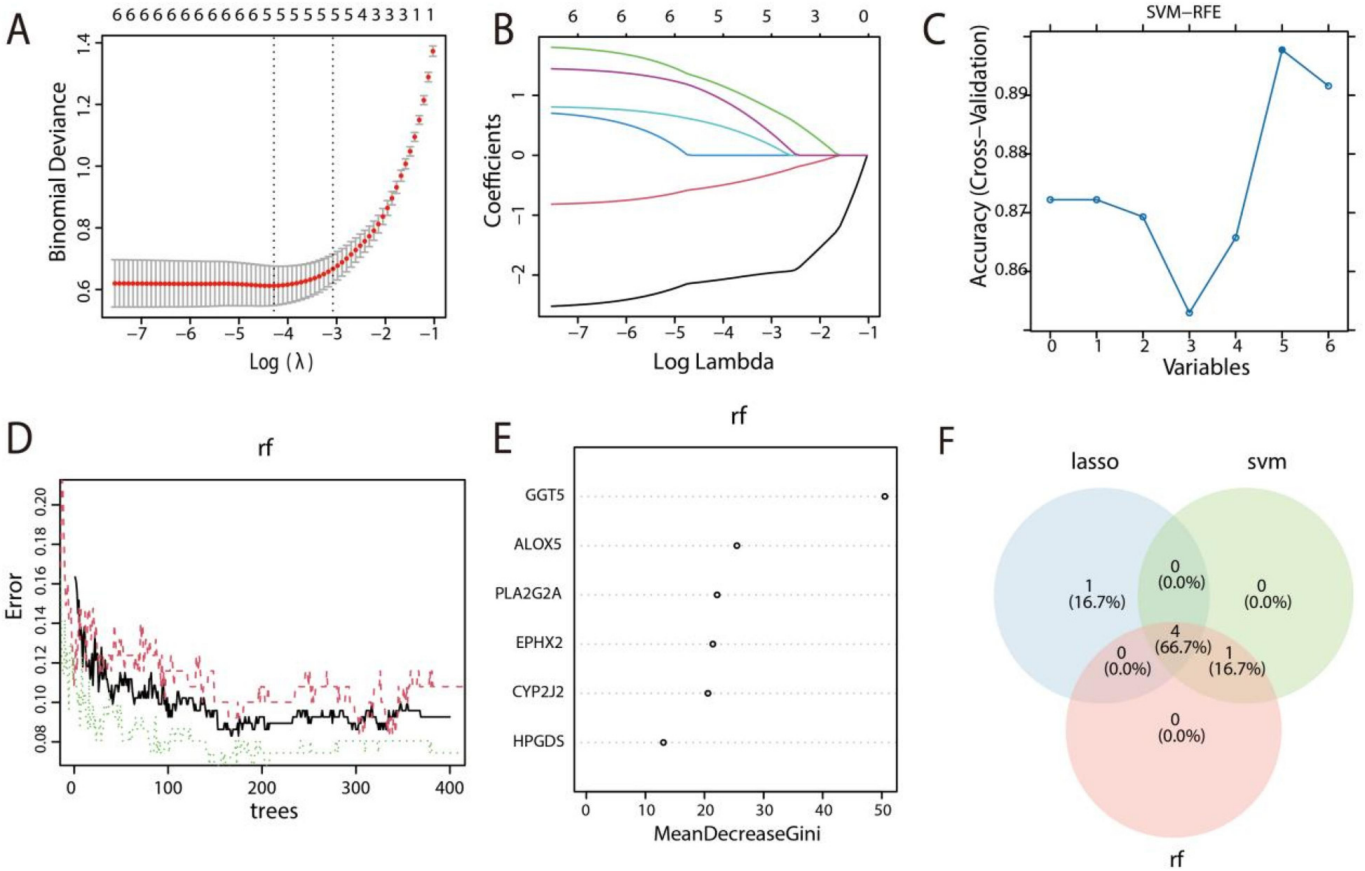
Identification of key DEAARGs associated with HF diagnosis. **(A)** Distribution of Coefficient along the logarithmic (*λ*) sequences within the LASSO regression model. **(B)** Coefficient spectrum of LASSO analysis. **(C)** Validation of signature gene expression based on SVM-RFE selection. **(D)** Residual distribution plot of the RF model, where the abscissa denotes the number of subtrees and the ordinate indicates the error. As the number of subtrees increases, the error decreases gradually. **(E)** Feature importance plot for RF. **(F)** Venn diagram illustrating the overlapping genes identified through the integrating of the three algorithms.

To identify prognostic feature genes for HF and filter out non-essential genes, the three machine learning algorithms, LASSO, SVM-RFE, and RF, were employed in this study. Firstly, five predictive genes were identified through LASSO regression analysis (Figures 3A,B). Thereafter, support vector machine-recursive feature elimination (SVM-RFE) was employed to identify 5 candidate genes (Figure 3C). Crucial genes were determined using RF analysis according to the MeanDecreaseGini criterion, helping to mitigate overfitting, which calculated the relationship across the error rate and the number of trees (Figure 3D), and the top 5 genes were then selected (Figure 3E). Finally, using three machine-learning algorithms (LASSO, RF, and SVM-RFE) with internal 10-fold cross-validation to ensure model stability, we identified four robust diagnostic markers (CYP2J2, EPHX2, PLA2G2A, and GGT5) that were consistently selected across all methods (Figure 3F).

### 3.3 Diagnostic performance evaluation and expression pattern analysis of feature genes

**Figure 4:**
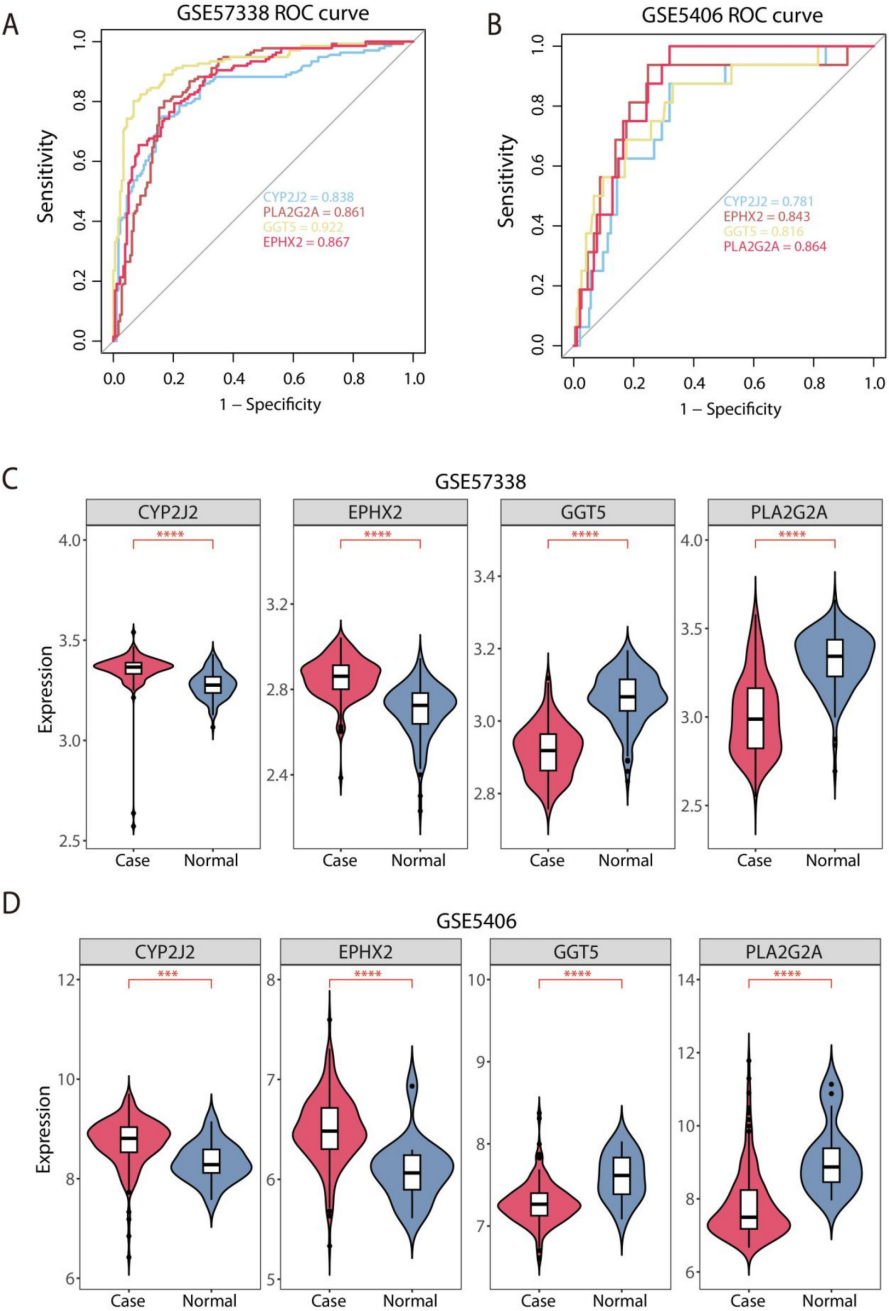
Assessment of diagnostic performance and expression profiles of feature genes. **(A)** Evaluation of the diagnostic performance of feature genes via ROC analysis in the GSE57338 dataset. **(B)** Evaluation of the diagnostic performance of feature genes via ROC analysis in the validation GSE5406 database. **(C)** Analysis of 4 feature gene expression profiles in the training set. **(D)** Comparative analysis of 4 feature gene expression profiles in the validation set. ***P < 0.001, and ****P < 0.0001.

The GSE57338 dataset was used as the internal training set to evaluate the diagnostic performance of the signature genes for HF, while the GSE5406 cohort was used for external validation. The AUC values of ROC curves plotted using “pROC” package in the training set were all greater than 0.8, indicating that the feature genes demonstrate excellent diagnostic performance, as reflected by the high sensitivity and specificity of the model (Figure 4A). Additionally, the AUC values of GGT5, PLA2G2A, EPHX2, and CYP2J2 were 0.781, 0.843, 0.816, and 0.864 in validation set GSE5406, confirming the strong diagnostic capability of the feature genes, validating the model's generalizability (Figure 4B). Meanwhile, the expression patterns of the 4 feature genes were also performed across the training set and the validation set. The box plots revealed that CYP2J2 and EPHX2 were significantly overexpressed in HF tissues, while GGT5 and PLA2G2A were significantly underexpressed in the training set GSE57338 (Figure 4C). These findings were consistent across the validation set, further corroborating the robustness of the feature genes as diagnostic biomarkers (Figure 4D).

### 3.4 Development and assessment of the HF diagnostic model

**Figure 5:**
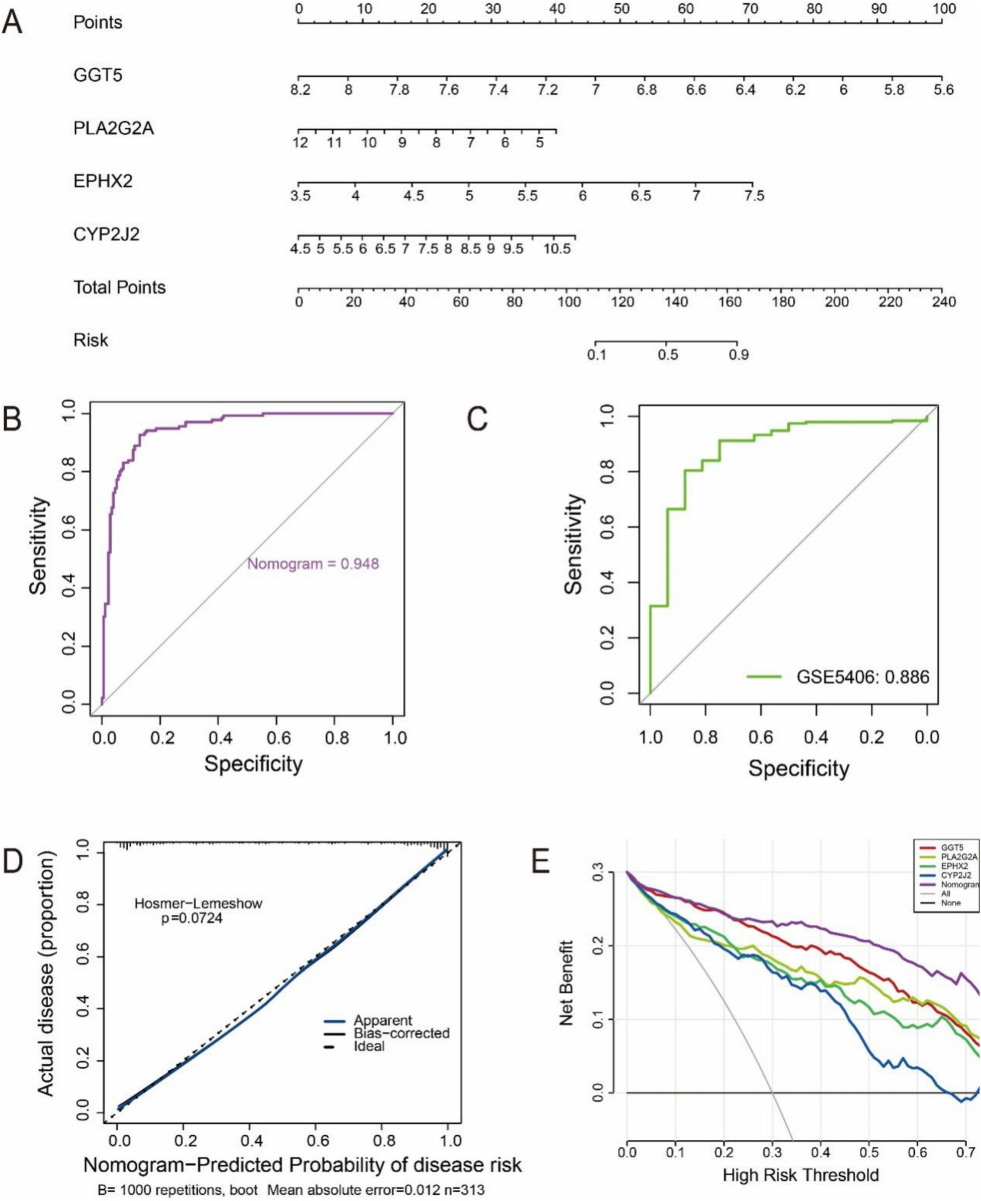
Establishment and assessment of the HF diagnostic model. **(A)** A comprehensive risk assessment nomogram. **(B)** ROC curve of the nomogram in the training cohort (GSE57338), showing an AUC of 0.948. **(C)** ROC curve in the external validation cohort (GSE5406), with an AUC of 0.886, confirming strong diagnostic performance and robustness of the model. **(D)** Outcomes of the calibration curve assessment. **(D)** Visualization of the DCA curve results.

To support clinical decision-making and improve risk assessment, we developed a comprehensive nomogram based on diagnostic feature genes. This nomogram enables rapid and accurate risk prediction for developing HF by calculating a patient's risk score (Figure 5A). Further, the AUC of ROC curves revealed excellent discrimination capability within the study dataset (Figure 5B). As shown in Figure 5C, the ROC analysis demonstrated that the nomogram maintained high discriminatory power in the validation dataset, achieving an AUC of 0.886, which was comparable to the performance observed in the training cohort. The calibration curves exhibited a strong alignment across predicted and observed probabilities, affirming the model's reliability in clinical applications (Figure 5D). DCA results revealed that robust diagnostic performance of both the feature genes and the nomogram over a broad spectrum of threshold probabilities, underscoring their prospective utility in clinical practice (Figure 5E). These results indicated that the diagnostic model based on the four characteristic genes demonstrated excellent predictive performance for HF.

### 3.5 Gene set enrichment analysis (GSEA) of feature genes

**Figure 6:**
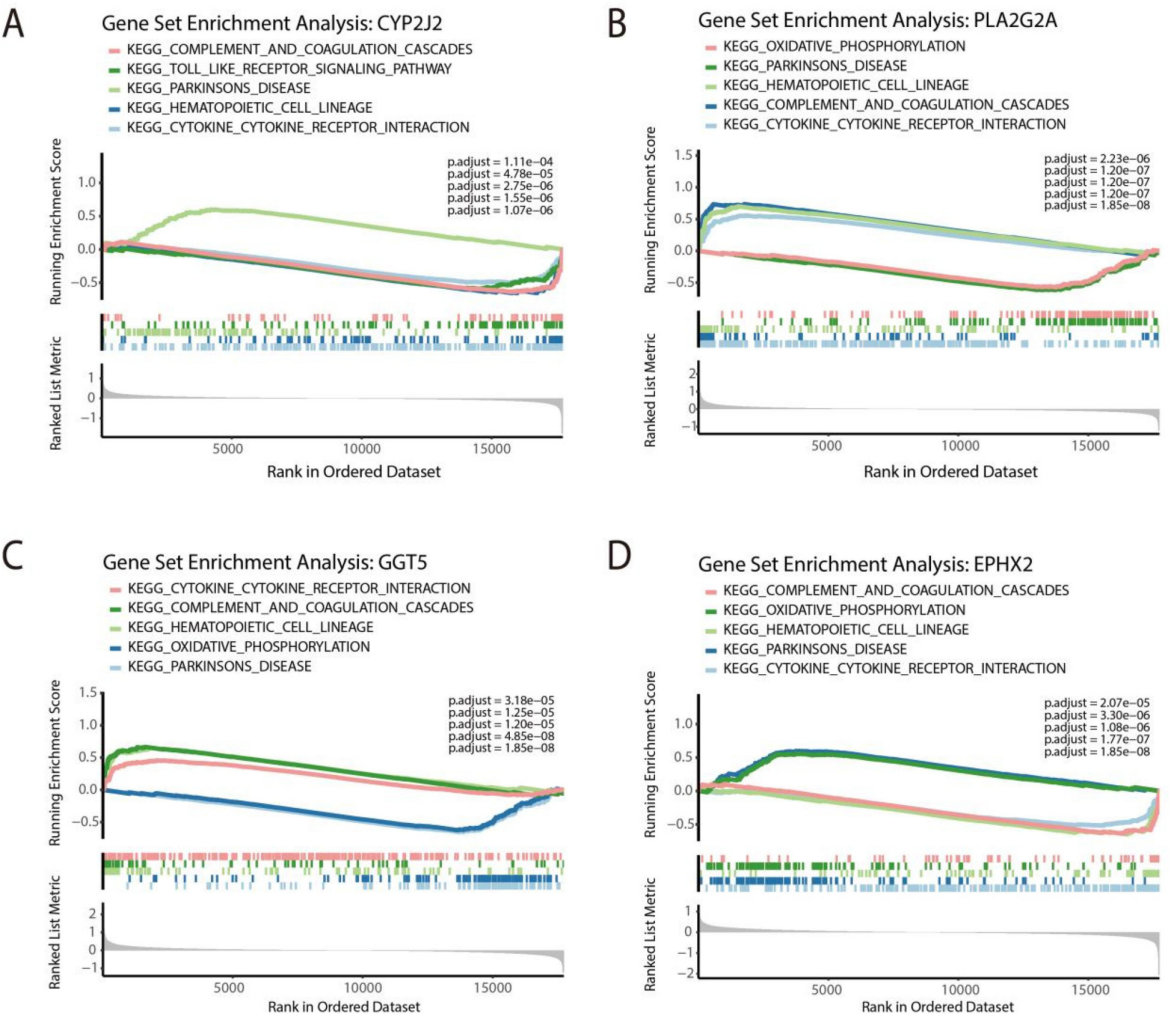
GSEA of feature genes. GSEA of CYP2J2 **(A)**, PLA2G2A **(B)**, GGT5 **(C)**, and EPHX2 **(D)** for top 5 pathways.

To investigate biological processes associated with each marker gene, we performed GSEA by stratifying samples into high- and low-expression groups based on the median expression of each feature gene. CYP2J2 was found to be negatively enriched in the following four pathways: complement and coagulation cascades, toll like receptor signaling pathway, hematopoietic cell lineage, and cytoking cytoking receptor interaction, and positively enriched in Parkinsons disease (Figure 6A). The gene of PLA2G2A was positively enriched within hematopoietic cell lineage, complement and coagulation cascades, and cytokine cytokine receptor interaction, and negatively enriched in oxidative phosphorylation, Parkinsons disease (Figure 6B). Furthermore, GGT5 displayed positive enrichment in three pathways, including cytokine cytokine receptor interaction, complement and coagulation cascades, and hematopoietic cell lineage, and negative enrichment in two pathways, including oxidative phosphorylation and Parkinsons disease (Figure 6C). On the other hand, EPHX2 showed positive enrichment in oxidative phosphorylation and Parkinsons disease and negative enrichment in complement and coagulation cascades, hematopoietic cell lineage and cytokine cytokine receptor interaction (Figure 6D).

### 3.6 Exploration of the immune landscape in HF

**Figure 7:**
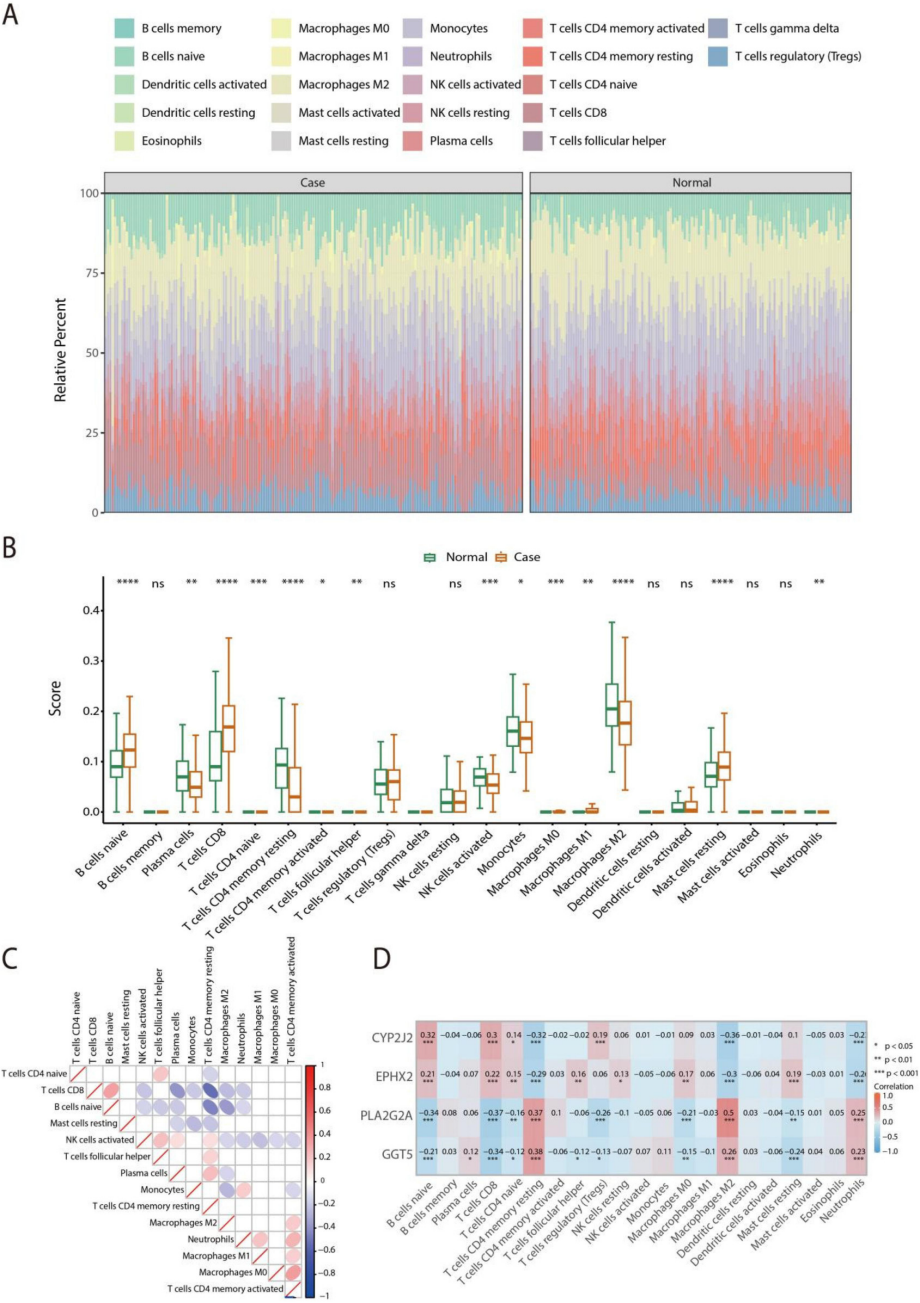
Assessment of immune cell infiltration in normal and HF samples using the CIBERSORT algorithm. **(A)** Comparative analysis of the infiltration levels of 22 immune cell types between normal and HF tissues. **(B)** The distribution of immune cell infiltration levels between normal and HF groups is shown via box plot analysis. **(C)** Correlation matrix illustrating the relationships among 14 immune cell types in normal and HF groups. **(D)** Spearman's rank correlation analysis was were calculated to explore the linkage between feature gene expression and immune infiltration. ns indicates non-significant differences, * signifies *P* < 0.05, ** denotes *P* < 0.01, and *** means *P* < 0.001.

To asses the immune cell infiltration landscape within the HF microenvironment, the CIBERSORT was applied to quantify the infiltration levels of 22 immune cell types in both normal and HF samples (Figure 7A). Box plot indicated that significant alterations were observed in 14 immune cell populations between the normal and HF groups, including B cells naive, Plasma cells, T cells CD8, T cells CD4 naive, T cells CD4 memory resting, T cells CD4 memory activated, T cells follicular helper, NK cells activated, Monocytes, Macrophages M0, Macrophages M1, Macrophages M2, Mast cells resting, and Neutrophils (Figure 7B). Further, the heatmap was conducted by Spearman analysis to examine the differential infiltration patterns of immune cells across the normal and HF samples (Figure 7C). Spearman correlation analysis showed that CYP2J2 and EPHX2 were positively correlated with B cells naive and T cells CD8, but negatively with macrophages M2. PLA2G2A and GGT5 were positively correlated with T cells CD4 memory resting and macrophages M2, and negatively with T cells CD8 (Figure 7D). These findings indicated distinct associations between the four hub genes and immune-cell subsets in HF.

### 3.7 Establishment of the ceRNA network for feature genes and prediction of potential drug targets

**Figure 8:**
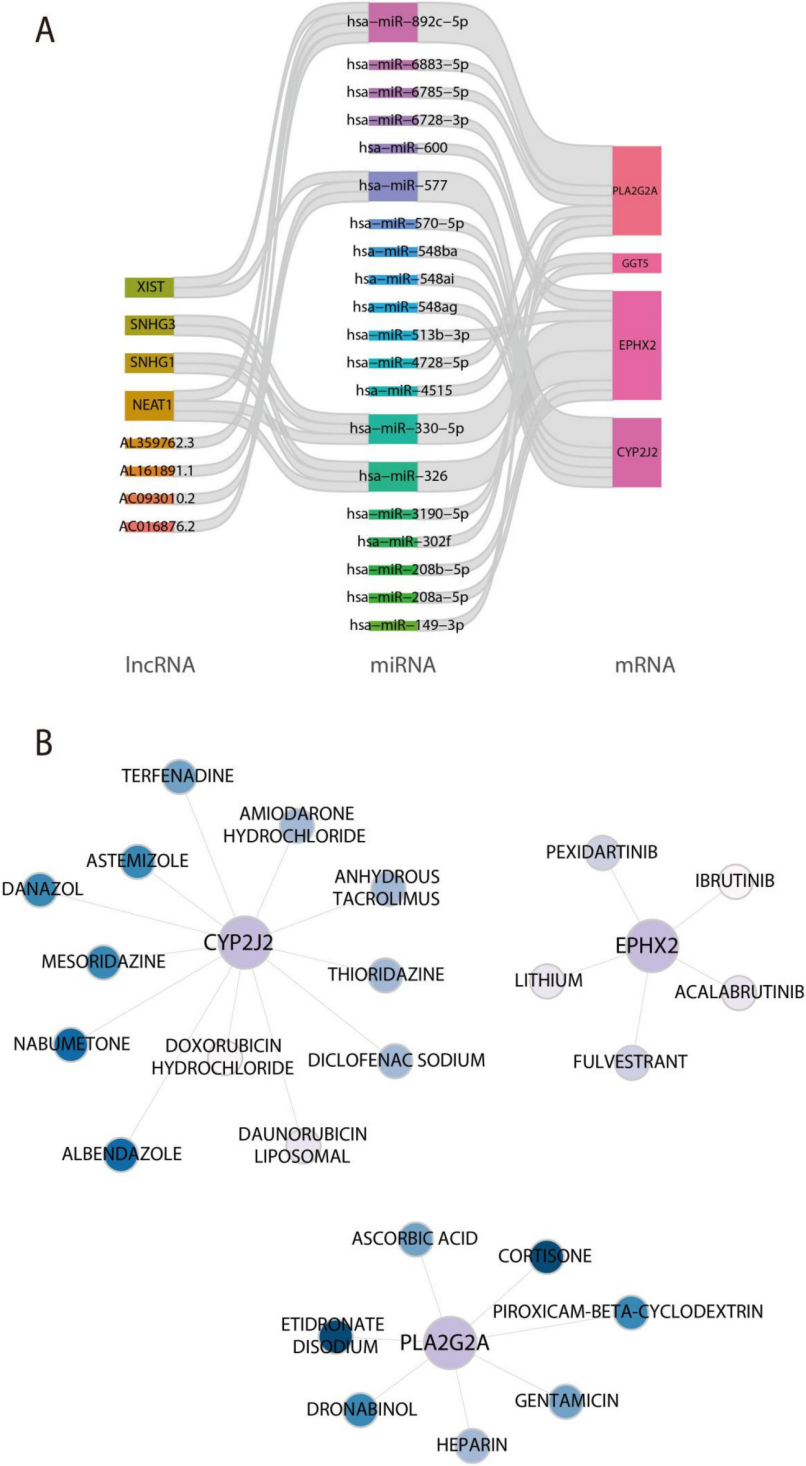
Establishment of the ceRNA regulatory network based on feature genes and prediction of potential drug targets. **(A)** The ceRNA network constructed based on feature genes. **(B)** Exploration of prospective drug candidates for CYP2J2, EPHX2, and PLA2G2A.

To elucidate the post-transcriptional regulatory mechanisms of feature genes, a ceRNA network was established (Figure 8A). The constructed ceRNA network includes 33 validated interactions, comprising 20 miRNAs, 8 lncRNAs and 3 mRNA. Subsequently, the signature genes-drugs networks were mapped to predict potential drugs for the characterised genes and selected approved drugs based on the DGIdb database (Figure 8B). These findings revealed that 12 approved drugs was predicted as potential drugs for CYP2J2, including Nabumetone, Albendazole, danazol and so on, 5 approved drugs was predicted as potential drugs for EPHX2, including Pexidartinib, Fulvestrant, and Acalabrutinib, 7 approved drugs was predicted as potential drugs for PLA2G2A, including etidronate Disodium, Cortisone, and Dronabinol.

### 3.8 Consensus clustering and immune infiltration analysis

**Figure 9:**
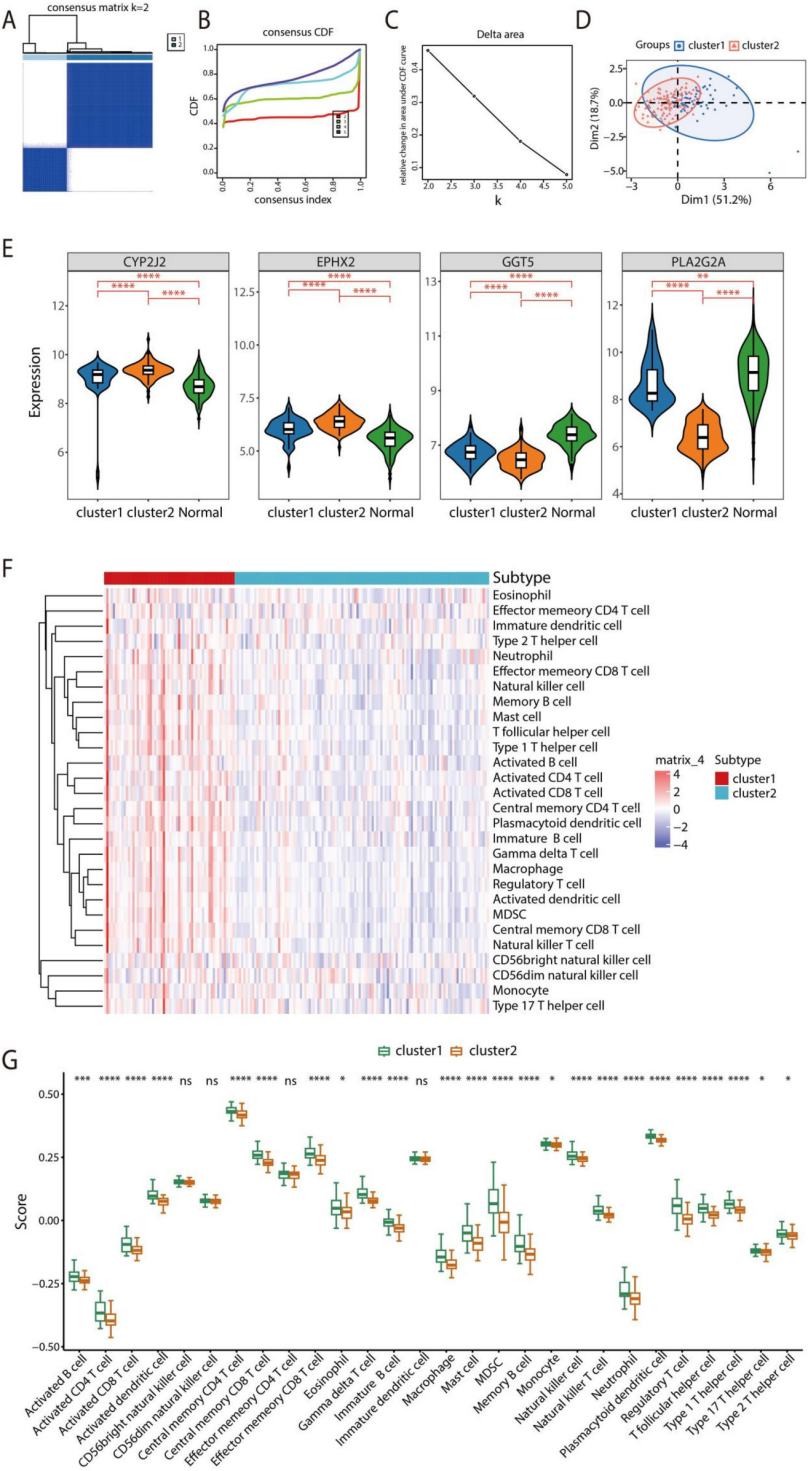
Consensus clustering and immune infiltration analysis. **(A)** Consensus Clustering matrix shows two molecular subtypes. **(B)** CDF across various cluster numbers. **(C)** Variation in the area under the curve (AUC). **(D)** PCA was conducted to differentiate across the two molecular subtypes. **(E)** The relationship between signature genes and immune cell infiltration levels among cluster 1, cluster 2 and normal samples. **(F)** Evaluation of immune cell infiltration across both clusters based on ssGSEA analysis. **(G)** Box plot illustrating immune infiltration across cluster 1 and cluster 2.

Consensus clustering analysis based on the expression profiles of feature genes was conducted using the “ConsensusClusterPlus” package. By assessing the cumulative distribution function (CDF) curve and its area, we determined the clustering as K = 2, dividing the HF samples into two distinct clusters (Figures 9A–C). Principal Component Analysis (PCA) analysis reliably and consistently polarized the samples into two directions (Figure 9D). The expression levels of the 4 signature genes, CYP2J2, EPHX2, GGT5, and PLA2G2A, were performed among cluster1, cluster2, and normal samples, revealing that CYP2J2 and EPHX2 exhibited elevated expression levels in the two clusters predominantly associated with normal samples, whereas GGT5 exhibited a decreased expression level. Notably, a lower expression of PLA2G2A was observed in cluster 2 compared with both cluster 1 and normal samples (Figure 9E). Additionally, ssGSEA was utilized to assess differences in immune cell infiltration across the two clusters (Figure 9F). The box plot revealed that the infiltration levels of 24 immune cell types were elevated in cluster 1 compared to cluster 2, including Activated B cell, Activated CD4 T cell, Activated CD8 T cell, Activated dendritic cell, Central memory CD4 T cell, Central memory CD8 T cell, Effector memeory CD8 T cell, Eosinophil, Gamma delta T cell, Immature B cell, Macrophage, Mast cell, MDSC, Memory B cell, Monocyte, Natural killer cell, Natural killer T cell, Neutrophil, Plasmacytoid dendritic cell, Regulatory T cell, T follicular helper cell, Type 1 T helper cell, Type 17 T helper cell, and Type 2 T helper cell (Figure 9G).

### 3.9 Experimental validation of feature genes by qRT-PCR

**Figure 10:**
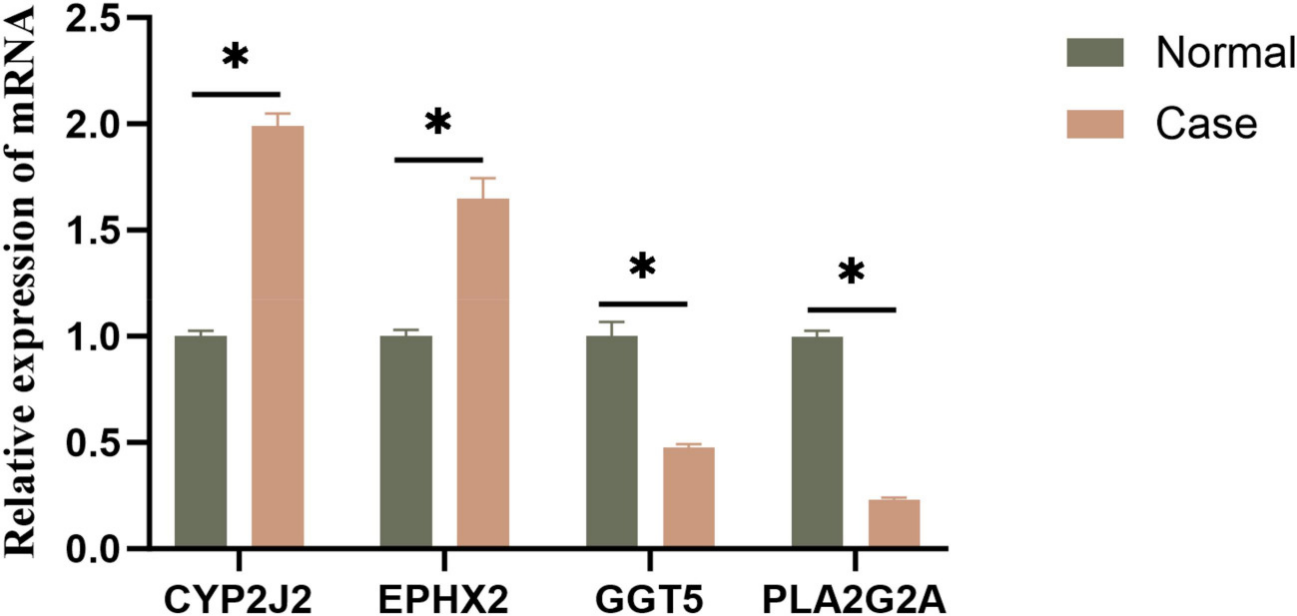
Experimental validation of feature genes by qRT-PCR. qRT-PCR analysis of CYP2J2, EPHX2, GGT5, and PLA2G2A.

To experimentally validate the signature genes identified in our analysis, qRT-PCR assays were performed. The analysis indicated that CYP2J2 and EPHX2 were found to be significantly upregulated in HF samples compared with normal samples, while GGT5 and PLA2G2A were significantly downregulated in HF samples (Figure 10). Hence, we speculated that the abnormal gene expression might play a pivotal role in driving HF.

## 4 Discussion

In this study, we systematically investigated the expression patterns and biological significance of AARGs in HF. Through integrative bioinformatics and machine-learning approaches, we identified four key feature genes—GGT5, PLA2G2A, EPHX2, and CYP2J2—strongly associated with HF. Immune infiltration analysis revealed significant alterations in multiple immune-cell subsets, suggesting immunometabolic crosstalk within the HF microenvironment. Furthermore, ceRNA network construction and drug-gene interaction prediction provided potential regulatory and therapeutic insights. Collectively, these findings enhance our understanding of the molecular mechanisms underlying AA metabolism dysregulation in HF and highlight potential biomarkers and drug targets.

We conducted a comprehensive analysis of DEGs between HF patients and normal controls from the GEO database, ultimately identifying six DEAARGs: PLA2G2A, ALOX5, CYP2J2, EPHX2, GGT5, and HPGDS. According to KEGG pathway analysis, the indicated DEAARGs were predominantly associated with the arachidonic acid metabolism pathway, implying their possible role in HF initiation and advancement. By integrating bioinformatics approaches with advanced machine learning algorithms, we identified four key hub genes: GGT5, PLA2G2A, EPHX2, and CYP2J2. Consistently, previous studies have reported that these four hub genes—CYP2J2, EPHX2, PLA2G2A, and GGT5—are closely associated with cardiovascular diseases (26–29). GGT5, a constituent of the γ-glutamyltransferase family, plays a role in the metabolism of glutathione, which is a critical pathway for cellular antioxidant defense (30). Recent studies have indicated that GGT5 overexpression can enhance endothelial function and reduce oxidative damage in vascular tissues (31). Given that oxidative stress and endothelial dysfunction are pivotal mechanisms underlying the progression of heart failure and other CVDs, it is plausible that GGT5 exerts a protective role in the cardiovascular system. Therefore, these findings are consistent with our results that the expression of GGT5 is elevated in normal samples compared to HF samples, which indicated that GGT5 may serve as a potential target for the diagnosis and therapy of HF. PLA2G2A, phospholipase A2 group IIA, has recently gained recognition for its critical involvement in the underlying mechanisms of CVDs (32). Wang et al. have revealed that PLA2G2A is associated with myocardial fibrosis by interacting with fibroblast-specific marker genes, a key process in heart failure progression (28). Additionally, experimental studies using atherosclerosis-prone mouse models demonstrated that genetic deletion of PLA2G2A significantly reduced plaque formation, highlighting its contribution to atherosclerotic development (33). These findings indicated that PLA2G2A may act as a potential biomarker or therapeutic target in heart failure and related conditions. Accumulating evidence indicates that EPHX2 (soluble epoxide hydrolase) is critically involved in the progression of CVDs) (34). In 2023, EPHX2 has been shown to enhance cardiac recovery following ischemia/reperfusion injury, suggesting its detrimental role in post-ischemic myocardial dysfunction (27). Genetic studies have further identified EPHX2 variants associated with heightened cardiovascular risk, such as aortic aneurysm and dissection, while the Lys55Arg polymorphism in EPHX2 correlates with increased long-term mortality after acute coronary syndrome (35, 36). CYP2J2, a specific type of cytochrome P450 epoxygenase, is predominantly expressed outside the liver and serves as the most crucial epoxygenase within the cardiac tissues of the human body (37). CYP2J2 is capable of converting AA into the regioisomers of cardioprotective epoxyeicosatrienoic acids, which is fundamentally involved in the protection and maintenance of cardiovascular system homeostasis (38). Further, reduced CYP2J2 expression has been related to disrupted ion channel regulation, extracellular matrix remodeling, and impaired energy metabolism in cardiomyocytes, processes associated with arrhythmias and HF progression (26). Therefore, the above findings suggest that these signature genes could represent potential therapeutic targets for HF.

Taken together, CYP2J2 and EPHX2, along with PLA2G2A and GGT5, appear to participate in interconnected stages of AA metabolism and oxidative regulation in HF. Within the cytochrome P450 branch, CYP2J2 serves as the principal cardiac epoxygenase that converts AA into EETs, which exert vasodilatory, anti-inflammatory, and anti-apoptotic effects (19). EPHX2, encoding soluble epoxide hydrolase (sEH), antagonizes this process by hydrolyzing EETs into less active dihydroxyeicosatrienoic acids (DHETs), resulting in diminished EET signaling and loss of cardioprotection (39). Additionally, PLA2G2A acts upstream by catalyzing the hydrolysis of membrane phospholipids to release AA, thereby providing substrates for downstream enzymatic pathways (40). GGT5, a γ-glutamyltransferase family member, regulates glutathione and eicosanoid conjugate metabolism, thereby influencing cellular redox homeostasis and inflammatory mediator turnover (29). Dysregulation among these nodes—characterized by reduced PLA2G2A and GGT5 expression together with elevated CYP2J2 and EPHX2—may reflect a disrupted AA-EET axis that favors oxidative stress, endothelial dysfunction, and maladaptive cardiac remodeling, ultimately contributing to HF progression (19). These findings suggest that coordinated modulation of AA metabolism, particularly through the CYP2J2/EPHX2 balance, could represent a promising therapeutic strategy for restoring metabolic and redox equilibrium in HF.

The immune microenvironment is increasingly recognized as an essential contributor to the pathogenesis of cardiovascular and metabolic conditions. According to a study by Zhou et al. (41), dysregulated immune responses involving macrophages, T cells, and B cells contribute to chronic inflammation, endothelial dysfunction, and tissue remodeling, thereby promoting atherosclerosis, myocardial infarction, and HF. Therefore, in this ecological network, where various components interact and influence each other, immune cells occupy a very important position (42). The assessment of immune cell infiltration indicated significant variations among immune cells, including Plasma cells, T cells CD4^+^ memory resting, NK cells activated, Monocytes, and Macrophages M2 were downregulated in HF samples compared with normal samples. Plasma cells are one of the cells that constitute immunological memory, working together with memory T cells and B cells to maintain a complex of sustained antibody titers, and produce large quantities of antibodies, playing a crucial role in immune protection (43, 44). Recent investigations have additionally underscored that the critical role of CD4⁺ T cells in the development and progression of CVDs and HF. A study has indicated that the increased circulating CD4⁺ T cell subsets, such as Th17 and T_EMRA cells, have been linked to higher cardiovascular risk (45). Furthermore, CD4^+^ T cells play a critical role in HF and CVDs by driving chronic inflammation and promoting myocardial fibrosis (46). According to another survey, CD4+ T cells can exert cardioprotective effects in preclinical models of acute myocardial infarction by acquiring the T regulatory phenotype and suppressing local inflammation, which consistent with out results (47). NK cells are immune cells with innate immunity, playing an important role in inflammation and immune responses (48, 49). Current research has shown that the characteristics of HF involved not only macrophages and T cells, but also NK cells and other immune cell subsets, making them new biomarkers for identifying HF and potential therapeutic targets (50). The progression of HF and CVDs is closely associated with monocyte activity.Recent research has reported that alterations in monocyte subsets contributed to systemic inflammation and cardiac remodeling in HF patients (51, 52). Macrophages M2 are closely associated with the inflammatory response, primarily involved in anti-inflammatory responses (53). In 2019, Peter et al. have already indicated the roles of monocytes and macrophages in the inflammatory and reparative phases following myocardial infarction (54).

Additionally, potential therapeutic agents targeting the identified feature genes were predicted using the DGIdb database, and several approved drugs were screened for CYP2J2, EPHX2, and PLA2G2A. To move beyond a simple drug list, these compounds were further evaluated in the context of their known pharmacological actions and potential relevance to the AA metabolic network. For CYP2J2, a cardiac AA epoxygenase generating cardioprotective EETs, the COX-inhibiting NSAID nabumetone may reduce prostanoid synthesis and divert AA flux toward the CYP epoxygenase branch, while danazol, an androgenic agent known to modulate CYP expression, could indirectly influence cardiac epoxygenase activity (19, 37). For EPHX2, which encodes soluble epoxide hydrolase responsible for EET degradation, the predicted drugs (pexidartinib, fulvestrant, and acalabrutinib) likely act through indirect transcriptional or pathway-level regulation rather than direct enzymatic inhibition, representing repurposing leads that warrant validation (55, 56). For PLA2G2A, which releases AA from membrane phospholipids, cortisone (a glucocorticoid) can suppress phospholipase A2 activity and thereby decrease AA availability, whereas dronabinol (a cannabinoid) may modulate endocannabinoid and AA-related signaling, offering a plausible anti-inflammatory link; etidronate shows only weak or indirect connections (57, 58). Collectively, the integration of gene-targeted drug prediction and ceRNA network analysis highlights a set of compounds with plausible mechanistic links to AA metabolism—through COX inhibition, PLA2 suppression, or modulation of the CYP/epoxide balance—and provides new avenues for optimizing drug selection and repurposing strategies in heart failure. Nevertheless, these predictions remain hypothesis-generating and require experimental verification.

Prior work has linked AA-related genes (CYP2J2, EPHX2, PLA2G2A, GGT5) to cardiac remodeling, but most studies were single-gene or pathway-specific, with modest cohorts and limited validation. In contrast, our AA-centered framework applies machine-learning feature selection, independent external validation, and integrates immune infiltration, ceRNA regulation, and AA-informed drug prioritization, providing a systems-level view. Strengths include multi-algorithm convergence and cross-dataset robustness with mechanistic context. Limitations include the retrospective use of public transcriptomes, potential inter-platform heterogeneity, and the need for protein-level and functional validation. These aspects define priorities for future mechanistic and translational studies.

Throughout this investigation, we employed three distinct machine learning techniques, enabling us to identify AARGs, including GGT5, PLA2G2A, EPHX2, and CYP2J2 as potential prognosis biomarkers for HF. Based on these genes, we developed a comprehensive nomogram to enhance precision therapy for patients with HF. Moreover, we also revealed the association of immune cell infiltration across normal and HF patients, as well as predicted potential therapeutic targets. Together, these findings collectively provide new insights into delivering personalised therapies with HF.

## 5 Conclusion

We focused on elucidating the potential link between AA and HF and identified four AARGs as diagnostic biomarkers of HF, which contributed meaningful perspectives on the prognostic evaluation of HF and laid a solid foundation for personalised treatment of HF patients. However, several limitations should be acknowledged in the present study, such as further *in vivo* and *in vitro* experiments are needed for validation.

## Data Availability

The original contributions presented in the study are included in the article/Supplementary Material, further inquiries can be directed to the corresponding author.
